# Influence of snow cover on albedo reduction by snow algae

**DOI:** 10.1128/mbio.03630-24

**Published:** 2025-01-14

**Authors:** Pablo Almela, James J. Elser, J. Joseph Giersch, Scott Hotaling, Trinity L. Hamilton

**Affiliations:** 1 Department of Plant and Microbial Biology, University of Minnesota, St. Paul, Minnesota, USA; 2 Flathead Lake Biological Station, University of Montana, Polson, Montana, USA; 3 Department of Watershed Sciences, Utah State University 4606 , Logan, Utah, USA; University of California, Irvine, Irvine, California, USA; University of Cambridge, Cambridge, United Kingdom

**Keywords:** snow algae, reflectance, subsurface

## Abstract

Snow algae darken the surface of snow, reducing albedo and accelerating melt. However, the impact of subsurface snow algae (e.g., when cells are covered by recent snowfall) on albedo is unknown. Here, we examined the impact of subsurface snow algae on surface energy absorption by adding up to 2 cm of clean snow to surface algal blooms and measuring reflectivity. Surprisingly, snow algae still absorb significant energy across an array of wavelengths when snow-covered. Furthermore, the scale of this effect correlates with algal cell densities and chlorophyll-a concentrations. Collectively, our results suggest that darkening by subsurface snow algae lowers albedo and thus potentially accelerates snowmelt even when the algae is snow-covered. Impacts of subsurface algae on melt await assessment. This implies that snow algae play a larger role in cryosphere melt than investigations of surface-only reflectance would suggest.

**IMPORTANCE:**

This study addresses a gap in research by examining the impact of subsurface snow algae on snow albedo, which affects snowmelt rates. Previous studies have focused on visible surface blooms, leaving the effects of hidden algae unquantified. Our findings reveal that snow algae beneath the surface can still absorb energy across various wavelengths, accelerating melt even when not visible to the naked eye. This suggests that spectral remote sensing can detect these hidden algae, although their biomass might be underestimated. Understanding how subsurface snow algae influence albedo and snowmelt is crucial for accurate predictions of meltwater runoff, which impacts alpine ecosystems, glacier health, and water resources. Accurate projections are essential for managing freshwater supplies for agriculture, drinking water, and other vital uses. Thus, further investigation into subsurface snow algae is necessary to improve our understanding of their role in snow albedo reduction and water resource management.

## OBSERVATION

Snow is the most reflective natural surface on Earth, reflecting >90% of visible radiation when freshly fallen (1). The primary determinant of snow’s albedo is its physical composition, primarily due to scattering at the interface between ice and air (2). However, the introduction of light-absorbing impurities (LAIs) reduces snow reflectance and enhances its absorption of solar energy. These impurities or contaminants can be abiotic (e.g., mineral dust) and biotic (e.g., algae). The influence of abiotic impurities on snow albedo has been thoroughly documented through field and remote sensing measurements (3). The effects of biological albedo reduction (BAR)—the collective influence of biological communities on albedo—are receiving increasing attention (4). Indeed, snow algae can reduce the albedo of the snow by ~17%–44% (5), likely making blooms of snow algae one of the largest global contributors to BAR (4).

Snow algae blooms dominate primary production on snow fields in alpine and polar ecosystems (6–8). The dominant algal taxa observed in this phenomenon are represented by genera *Chlamydomonas*, *Chloromonas*, and *Sanguina* (4) that color the snow green or red due to the production of the carotenoid astaxanthin (9, 10). The production of this pigment protects the cell against high UV radiation and may contribute to generating meltwater in the surrounding ice crystals by releasing heat from absorbed sunlight (11). Consequently, an increased abundance of snow algae increases rates of melting (8).

Snow algae predominantly accumulate on the surface of melting snow, but they can also manifest below the surface (1), either due to subsurface growth or when surface blooms become buried by fresh snowfall (e.g., in early fall). The vertical distribution of snow algae within the snowpack is likely an important factor in determining their impact on albedo. However, research that links snow algae to changes in albedo typically focuses on visible surface blooms (8, 12). Thus, in contrast to other LAIs (3, 13), the effects on albedo reduction when snow algae are found beneath the surface have not been quantified.

In this study, we investigated the effects of subsurface snow algae on snow reflectivity. We measured the hemispherical-directional reflectance factor (HDRF) across six plots within the same snow patch, each displaying different cell densities. While HDRF is a directional measure of reflectance, assessment of HDRF allows for a more controlled study of a specific surface of the snow, providing insights into potential changes in albedo. To examine the impact of snow cover on snow algae, we applied successive layers of clean snow from a nearby area in 0.5 cm layers up to 2 cm. We then measured reflectance for increasing depths of overlying clean snow (Fig. S1). Following this, we collected the biomass for analysis, measuring cell densities and chlorophyll-a concentrations (see Supplemental Information for details).

Samples were taken from plots exhibiting different color intensities, with cell densities ranging from 35,000 to 210,500 cells mL^−1^ (Table S1). This suggests a heterogeneous distribution of algae within the snow algae bloom. These biomass levels measured are comparable to the findings reported in reference 12 in the North Cascades but higher than those reported in reference 5 in the Arctic, suggesting large variability in cell densities in snow algae blooms on a global scale. Cell density and chlorophyll-a concentration were strongly correlated (*R* = 0.79, *P* < 0.05). This finding is relevant as chlorophyll quantity within an individual cell varies between species and can change over time as a photoacclimation mechanism (14). Hence, the association between pigmentation and cell abundance indicates the suitability of both methods in assessing biomass concentration within this snow algae bloom.

**Fig 1:**
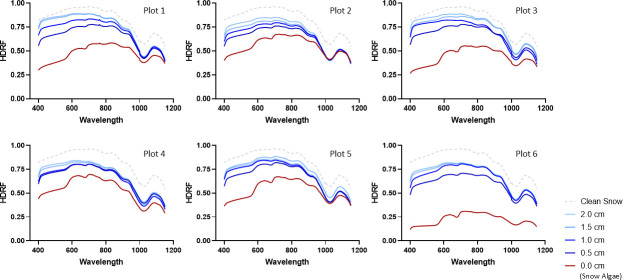
Variations in HDRF values (400–1,150 nm) measured on the different plots across a snow algae bloom after the various layers of added snow. Lines represent the mean of three replicates. The spectral albedo of clean snow was measured once and serves as a reference applied to all measurements.

Snow algae absorb light energy primarily in the ranges where their specific pigments demonstrate high efficiency in absorption. Across the spectrum of wavelengths we measured (400–1,150 nm), we observed a significant negative correlation between algal biomass and HDRF (−0.92 and −0.72 for cell density and chl-a, respectively; *P* < 0.05), demonstrating a sizable BAR effect. HDRF measurements of the snow algae also showed a strong reflectance decrease within the 400–580 nm range (carotenoids) and 600–700 nm range (chlorophylls) compared to clean snow (Fig. 1). For these absorption spectra (Table S1), we observed an expected significant negative correlation between cell density and light reflectance for carotenoids (−0.83, *P* < 0.05) and chlorophylls (−0.86, *P* < 0.01). Similar findings were noted in relation to chl-a concentrations (−0.68, *P* < 0.05 and −0.76, *P* < 0.05, respectively). This aligns with prior research (15) and indicates substantial light absorption by algal cells. Collectively, these results show that the radiative influence of snow algae is greatest in the visible region, where pigments efficiently absorb light but also occurs in the near-infrared region of the solar spectrum.

**Fig 2:**
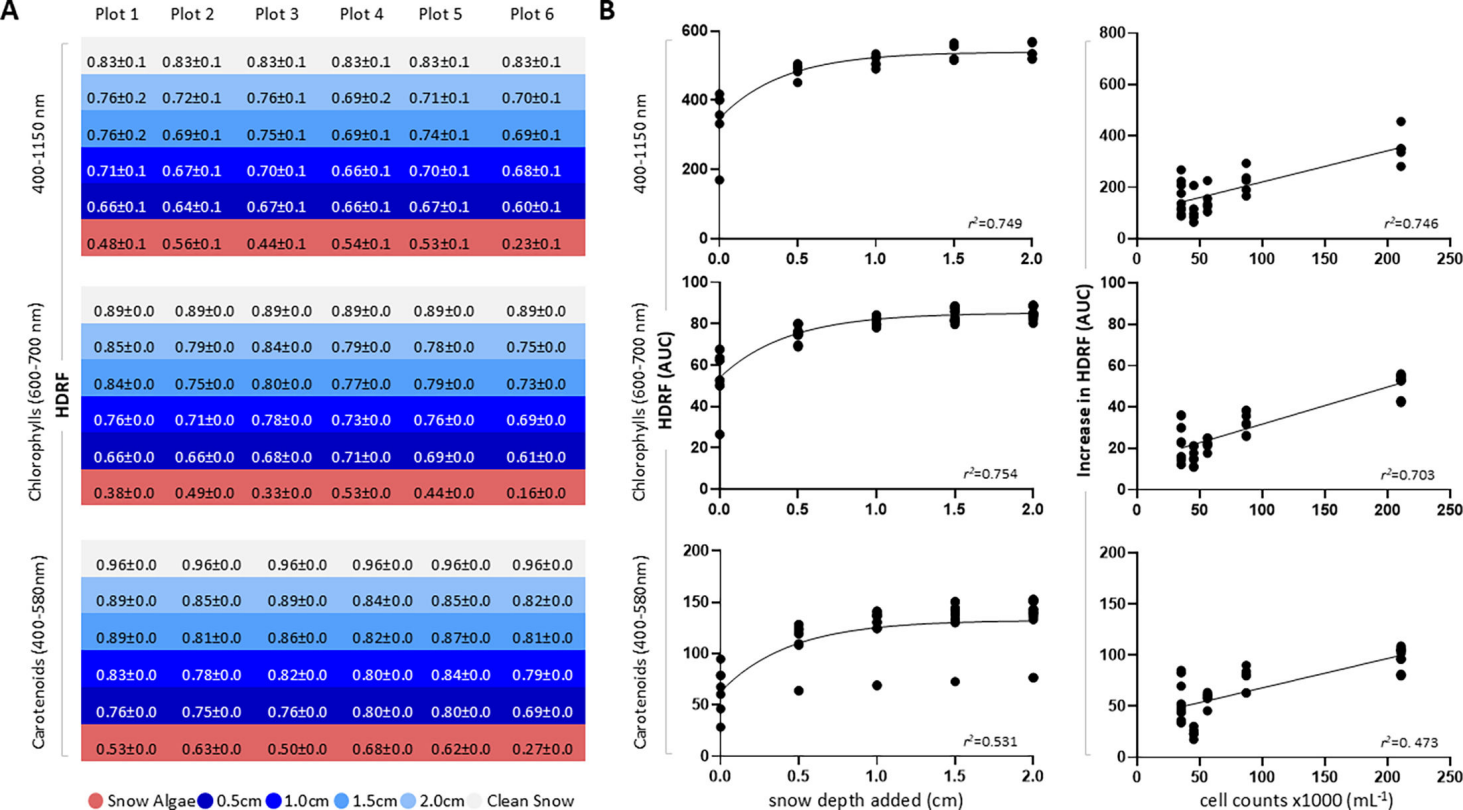
(**A**) Mean reflectance (HDRF) and standard deviation of the snow algae bloom and across the various snow layers added within the specified spectral albedo ranges (400–1,150 nm, 600–700 nm, and 400–580 nm). At the top of each wavelength range, the reflectance for the clean snow reference is also indicated. (**B**) Scatterplots of the regression analysis between the area under the curve (AUC) values of HDRF across the different spectra and the depths of snow layers and cell densities. Increase in HRDF was calculated by comparing the reflectance of each additional snow layer with that of the snow algae in the plot.

When we assessed how the HDRF of snow algae between 400 and 1,150 nm (Fig. 1) related to the depth of the snow added, we observed an increase in reflectance with depth of overlying snow for all plots (*P* < 0.05). The same results were obtained for the absorption ranges of carotenoids (*P* < 0.05) and chlorophylls (*P* < 0.05). A positive correlation fitting a logarithmic growth curve was found between HDRF and the thickness of the snow cover across the examined wavelength spectra (Fig. 2B). This relationship likely reflects an initial increase in light reflectance with added snow thickness, with a diminishing effect as depth increases. The absorption spectrum of chlorophylls displayed a stronger correlation with HDRF compared to carotenoids (with *r*^2^ values of 0.75 and 0.53, respectively). However, snow algae on the surface reflected more energy in the 400–580 nm range compared to 600–700 nm range (mean HDRF values of 0.54 ± 0.15 and 0.39 ± 0.13, respectively) (Fig. 2A). Green snow algae, with higher chlorophyll content than red snow algae, demonstrated increased solar radiation absorption, resulting in a greater reduction in albedo for an equivalent algae concentration in the snow (16). Our data indicate that chlorophylls exhibit higher energy absorption efficiency than carotenoids when covered by snow, and thus chlorophylls are more influential in determining how much light the snow algae reflect.

Collectively, these findings show that the presence of snow cover significantly influences the overall energy balance and radiative properties of snow algae blooms, highlighting a pronounced increase in reflected energy in the initial millimeters of snow added on the algae, which progressively decreases as the snow thickness increases. Increased HDRF was positively associated with snow algal biomass (*r*^2^ = 0.75 for HDRF measurements between 350 and 1,150 nm) (Fig. 2B). This suggests that the influence of snow cover on the reflectance of a snow algae bloom depends on the concentration of algae present. The HDRF for the 2.0 cm experimental snow layer added on the snow algae was lower compared to that seen in clean snow (Fig. 2A), showing significant differences in the studied spectral ranges (*P* < 0.05). These findings reveal that energy absorption occurs across all analyzed wavelengths even when snow algae are covered by snow up to 2.0 cm. While LAIs other than algae could exist in the analyzed snow, they were not quantified. Nevertheless, we assumed their effect would remain consistent in both clean snow and the experimental snow layered over the snow algae, thereby not compromising the validity of our conclusions. The efficiency of snow algae in absorbing sunlight may be crucial for sustained energy capture, enabling snow algae to thrive under lower-light conditions but also, potentially, to accelerate melting rates that sustain liquid water for nutrient uptake and growth. This indicates a link between snow algae and melting, even when algae are located beneath the snow surface and are visually undetectable. However, direct evidence of impacts on melting is required to confirm this relationship. Future studies may delve further into this phenomenon, and explore the impact of algae blooms with abundant pigmented biomass deeper in the snowpack.

Our results also have implications for remote sensing of snow algae and its effects on albedo. Efforts to use remote sensing for the identification and quantification of snow algae have increased in recent years, making it an effective tool for analyzing the temporal evolution of snow algae blooms at a regional scale (16, 17). However, detecting subsurface snow algae in visible range scans could be challenging as the snow cover attenuates the absorption of pigments. Additionally, snow algae may remain undetected by direct measurements and ground-based methods typically required for precise, detailed analysis and data validation (18). Therefore, explicit efforts to sample and quantify subsurface snow algae in these assessments should be considered. In cases where remote sensing proves insufficient for detection, studying snow algae impact on BAR by ground-based methods will be crucial for understanding their impact.

## SUPPLEMENTAL MATERIAL

Figure S1 mbio.03630-24-s0001.tif Spectral albedo assessment of a snow algae bloom using a spectroradiometer and a pistol grip optical fiber device.

Figure S2 mbio.03630-24-s0002.tif Variations in HDRF values measured on snow with and without the PVC cylinder used in this experiment.

Supplemental Methods mbio.03630-24-s0003.docx Additional experimental details.

Table S1 mbio.03630-24-s0004.docx Volumes, cell density, chlorophyll concentrations, and HDRF across the specific wavelength ranges analyzed for each sample in this study.

ASM does not own the copyrights to Supplemental Material that may be linked to, or accessed through, an article. The authors have granted ASM a non-exclusive, world-wide license to publish the Supplemental Material files. Please contact the corresponding author directly for reuse.
