# Supplementary figures and images for: Influence of snow cover on albedo reduction by snow algae
Source: mBio. 2025 Jan 14;16(2):e03630-24. doi: 10.1128/mbio.03630-24 (PMC11796388; doi:10.1128/mbio.03630-24)

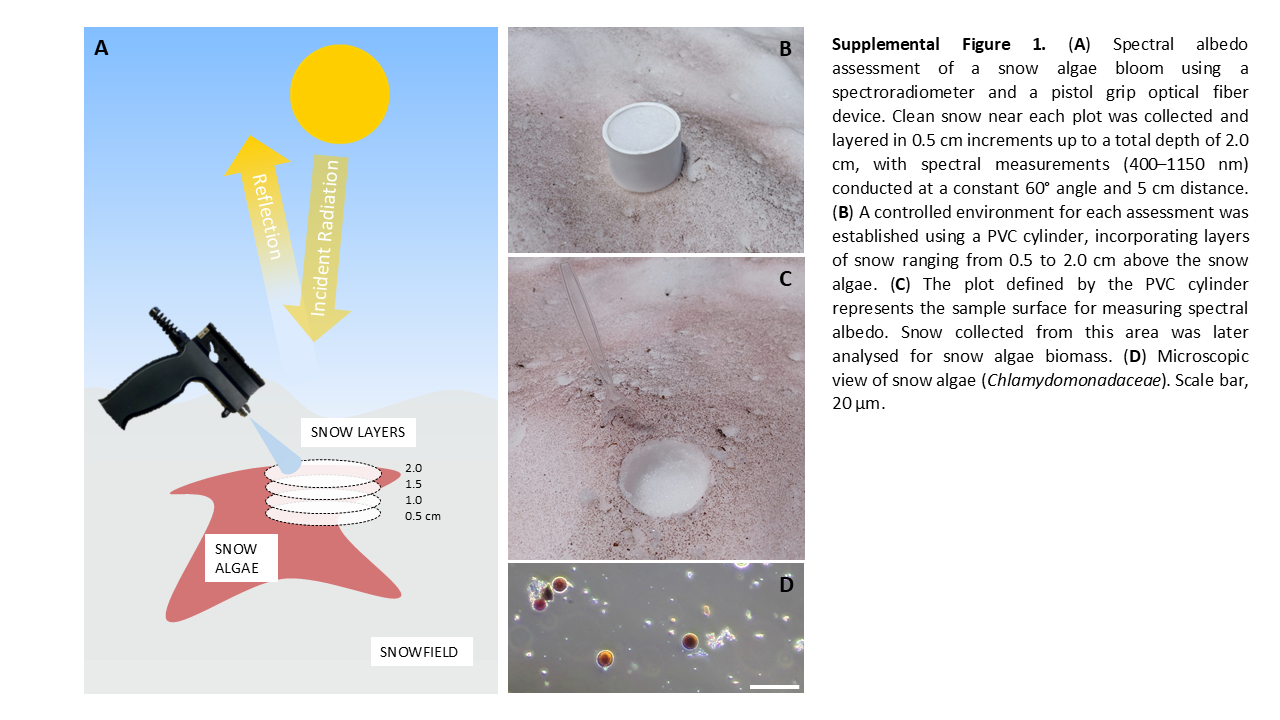

Supplement: Figure S1 — Spectral albedo assessment of a snow algae bloom using a spectroradiometer and a pistol grip optical fiber device. [file mbio.03630-24-s0001.tif]

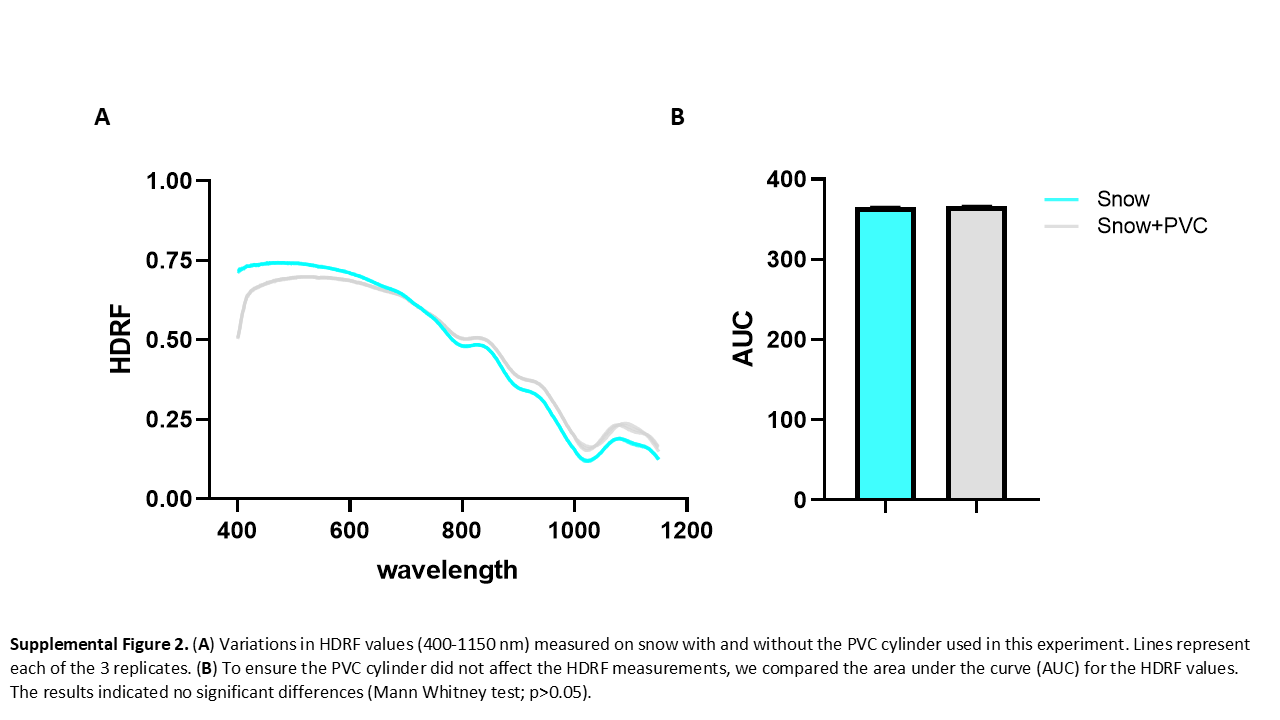

Supplement: Figure S2 — Variations in HDRF values measured on snow with and without the PVC cylinder used in this experiment. [file mbio.03630-24-s0002.tif]
