# Supplementary material for: Influence of snow cover on albedo reduction by snow algae
Source: mBio. 2025 Jan 14;16(2):e03630-24. doi: 10.1128/mbio.03630-24 (PMC11796388; doi:10.1128/mbio.03630-24)
Supplement: Supplemental Methods — Additional experimental details. [file mbio.03630-24-s0003.docx]

MATERIAL AND METHODS

### *Study Site*

Glacier National Park (GNP), referred to as *Ya·qawiswit̓xuki* ("the place where there is a lot of ice") by the Kootenai tribe, is in northwest Montana, United States. The Park preserves one of the most ecologically intact temperate regions of the world. During the Little Ice Age, an estimated 146 glaciers were within the current boundaries of GNP. Only 51 of these glaciers persisted until 2005 (1).

The experiment was conducted on a seasonal snowfield located at the northeast base of Clements Mountain (48°41'33" N 113°44'10" W) at Logan Pass. The snowfield, approximately 0.6 km^2^, had a slope of 20-22° and a snow depth ranging from 50-125 cm. The presence of snow algae was easily discernible from a distance (~100 m). To control for variation in the presence of snow algae and other factors, we replicated our experiment across six plots, spaced at least 2 m apart.

### *Measurements of spectral reflectance*

On 1 August 2023, HDRF of the six study plots was measured using a Analytical Spectral Devices (ASD) FieldSpec® 4 hyperspectral spectroradiometer (Malvern Panalytical, USA) with a spectral resolution of approximately 3 nm at 700 nm and 10–12 nm between 900 and 2500 nm. While the hyperspectral spectroradiometer measures between 350 to 2500 nm, the data presented in this study is limited to the spectral range of 400-1150 nm, due to the biological significance of this range and avoiding unnecessary extension into the far-infrared. We used a pistol grip device that allows a directional measurement with a field of vision of α = 25° (**Figure S1A**). Prior to measurements, a spectralon panel was used to perform a white reference measurement. Since snow and ice albedo is sensitive to the direction of incoming solar irradiance, all measurements were taken consecutively on the same day (between 1:00 PM and 3:00 PM) under the same conditions: clear-sky, facing the sun, and with a constant measurement angle of 60° and a distance between the optical fiber and a target of 5 cm (measured area ≈ 59.05 cm^2^).

To assess the influence of snow cover on the reflectivity of snow algae, a PVC cylinder (7.6-cm radius) was placed on the surface of the algae bloom to control the thickness of the snow layers added. A laboratory test was conducted to verify that the PVC cylinder did not affect the HDRF measurements by comparing the reflectance of snow with and without the PVC cylinder (**Figure S2**). After placing the PVC cylinder, an initial measurement of surface reflectance was made to measure the HDRF of the snow algae. Next, subsurface snow (visually brighter and free of apparent abiotic or biotic contaminants affecting albedo) was collected near each plot using a plastic scoop. This snow was then sequentially arranged in layers of 0.5 cm each, eventually reaching a total depth of 2.0 cm (**Figure S1B**). Based on our observations in real-time in the field at this snowfield, 2 cm seemed like a reasonable depth, a depth that is also consistent with the model described in (2).

Reflectance measurements (i.e., HDRF) were repeated in triplicate after adding each successive 0.5-cm layer. We also measured the HDRF of snow in an area without visible biotic or abiotic impurities after removing the top centimeter of snow to obtain a reference for the maximum attainable sunlight reflection within the surveyed snowfield at that time. We assumed that the physical characteristics of the snow were consistent across the sampled plots during the sampling period. Because carotenoids (absorbing in the 400-580 nm range) and chlorophylls (absorbing in the 600-700 nm range) distinctly influence the albedo of algae-containing snow (3), these specific wavelength ranges were chosen as optimal for investigating how specifically algae affect snow reflectivity, which in turn allows us to infer its effect on albedo reduction, across varying snow depths.

### *Sample collection, chlorophyll-a analysis, and cell counts*

Following HDRF measurements for each plot, the snow within the PVC cylinder (**Figure S1B**) and a 2-cm deep core, which includes the snow algae and aligns with the surface where reflectance measurements were conducted (**Figure S1C**) were both collected. The snow was placed in a sterile plastic bag and transferred to the laboratory for analysis.

Two approaches were used to estimate algal biomass to account for the variability of algal abundance present within the study snowfield (4). Of the total volume of the sample (~95 mL), a 100-µL aliquot was used to count cells, and the rest of the volume was filtered onto ashed 0.7-µm pore size Whatman^TM^ GF/C filters. Filters for chlorophyll analysis were extracted overnight in 90% acetone for fluorometric analysis using the acid-correction method (EPA Method 445.0) on a Turner 10-AU Fluorometer (with Optical Kit #10-037R). Cell counts were conducted the same day of sampling using a counting chamber (Hausser Scientific) and a light microscope (Leitz LaborLux S, with 10x objective).

The final concentrations of chlorophyll-a and cell density for each plot were calculated considering the final sample volumes and a constant volume of snow added on top of each plot (assuming a snow density of 500 kg m^-3^).

### *Statistical analysis*

We used Pearson correlations to assess relationships between biological and physical parameters of the snow. Statistically significant effects of increasing snow layer depth were determined with an ANOVA test. To better understand the relationships between snow cover and HDRF, and algae biomass and increase in HDRF, linear regression analysis was performed. The HDRF values were compared by calculating the area under the curve (AUC) across the absorption features present in the different spectra (e.g., 400-1150, 400-580 and 600-700). The increase in HRDF was calculated by comparing the reflectance of each snow layer added with the reflectance measured for the snow algae in the plot.

FIGURE CAPTIONS

Supplemental Figure 1. (A) Spectral albedo assessment of a snow algae bloom using a spectroradiometer and a pistol grip optical fiber device. Clean snow near each plot was collected and layered in 0.5 cm increments up to a total depth of 2.0 cm, with spectral measurements (400–1150 nm) conducted at a constant 60° angle and 5 cm distance. (B) A controlled environment for each assessment was established using a PVC cylinder, incorporating layers of snow ranging from 0.5 to 2.0 cm above the snow algae. (C) The plot defined by the PVC cylinder represents the sample surface for measuring spectral albedo. Snow collected from this area was later analysed for snow algae biomass. (D) Microscopic view of snow algae (*Chlamydomonadaceae*). Scale bar, 20 μm.

Supplemental Figure 2. (A) Variations in HDRF values (400-1150 nm) measured on snow with and without the PVC cylinder used in this experiment. Lines represent each of the 3 replicates. (B) To ensure the PVC cylinder did not affect the HDRF measurements, we compared the area under the curve (AUC) for the HDRF values. The results indicated no significant differences (Mann Whitney test; p>0.05).

Supplemental Table 1. Volumes, cell density, chlorophyll concentrations, and HDRF across the specific wavelength ranges analysed for each sample in this study.

REFERENCES

1. Martin-Mikle CJ, Fagre DB. 2019. Glacier recession since the Little Ice Age: Implications for water storage in a Rocky Mountain landscape. Arct Antarct Alp Res 51:280–289.
2. Onuma Y, Takeuchi N, Tanaka S, Nagatsuka N, Niwano M, Aoki T. 2020. Physically based model of the contribution of red snow algal cells to temporal changes in albedo in northwest Greenland. Cryosphere 14:2087–2101.
3. Painter TH, Duval B, Thomas WH, Mendez M, Heintzelman S, Dozier J. 2001. Detection and quantification of snow algae with an airborne imaging spectrometer. Appl Environ Microbiol 67:5267–5272.
4. Cook JM, Hodson AJ, Gardner AS, Flanner M, Tedstone AJ, Williamson C, Irvine-Fynn TDL, Nilsson J, Bryant R, Tranter M. 2017. Quantifying bioalbedo: a new physically based model and discussion of empirical methods for characterising biological influence on ice and snow albedo. Cryosphere 11:2611–2632.
