# Supplementary material for: Influence of snow cover on albedo reduction by snow algae
Source: mBio. 2025 Jan 14;16(2):e03630-24. doi: 10.1128/mbio.03630-24 (PMC11796388; doi:10.1128/mbio.03630-24)
Supplement: Table S1 — Volumes, cell density, chlorophyll concentrations, and HDRF across the specific wavelength ranges analyzed for each sample in this study. [file mbio.03630-24-s0004.docx]

| Sample | Sample Volume (mL) | Cells/ml | Chl-a  (µg/L) | HDRF 400-1150 nm  (AUC) | HDRF 400-580 nm  (AUC) | HDRF 600-700 nm  (AUC) |
| --- | --- | --- | --- | --- | --- | --- |
| 1 | 31.5 | 35,000 | 16.20 | 357.7 | 67.58 | 52.69 |
| 2 | 31.8 | 45,000 | 29.70 | 418.3 | 87.82 | 63.48 |
| 3 | 35.8 | 87,000 | 25.20 | 332.4 | 60.27 | 50.18 |
| 4 | 33.3 | 35,000 | 7.20 | 401.8 | 94.6 | 67.63 |
| 5 | 39.5 | 56,000 | 7.65 | 399.7 | 78.82 | 62.35 |
| 6 | 42.3 | 210,500 | 56.70 | 169.8 | 28.39 | 26.56 |

**Supplemental Table 1**. Volumes, cell density, chlorophyll concentrations, and HDRF across the specific wavelength ranges analysed for each sample in this study.
